# Leaf water content and water source partitioning reveal species-specific drought vulnerabilities in subtropical shrubs

**DOI:** 10.3389/fpls.2025.1684521

**Published:** 2025-10-28

**Authors:** Wenxi Peng, Bo Jiang, Leru Chang, Zidong Luo

**Affiliations:** ^1^ School of Economics and Management, Hunan Open University, Changsha, China; ^2^ Department of Eco-culture and Eco-Tourism, Hunan Vocational College of Engineering, Changsha, China; ^3^ Institute of Subtropical Agriculture, Chinese Academy of Sciences, Changsha, China; ^4^ Guangxi Key Laboratory of Karst Ecological Processes and Services, Huanjiang Observation and Research Station of Karst Ecosystems, Chinese Academy of Sciences, Huanjiang, Guangxi, China

**Keywords:** soil water content, stable isotopes, drought vulnerability, leaf water status, wateruptake depth

## Abstract

Hydraulic regulation of leaf water content and root water uptake underpins drought resistance in woody plants, yet these processes remain poorly quantified in humid forest shrubs. Here we explored drought vulnerability of two shrubs in a subtropical humid forest based on field measurements of soil water content (SWC), leaf water content (LWC), and isotopic compositions (δ^2^H and δ^18^O) of xylem and soil water. The results showed that during the drought in 2022, SWC within the 0–100 cm depth declined sharply, with severe soil water deficiency persisted for more than three months. Consequently, the two shrubs exhibited significant differences in LWC over time. During the drought, LWC declined 22.1% in *L. polystachyus* versus a more pronounced 35.2% drop in *V. negundo* compared to wet periods. Meanwhile, LWC was a useful predictor of drought vulnerability and exhibited a threshold-type response that distinguished individual plants at no risk from those at increasing risk of drought-induced canopy damage/dieback. Water stable isotopes revealed that *L. polystachyus* and *V. negundo* both mainly rely on shallow (0–30 cm) soil water (accounting for 58.8 and 70.5%, respectively) during wet period. However, it showed fundamentally divergent drought-response strategies during drought period: *L. polystachyus* enhanced drought resistance through progressive deep-water foraging (from 12.6% to 19.9%), while *V. negundo* maintained greater reliance on ephemeral shallow resources (accounting for 66.6%) and was thus more vulnerable. In addition, differences in SWC and plant size were important factors influencing plant water status and drought responses. These findings provide a useful framework to evaluate species differences in drought vulnerability regulated by water resource acquisition and plant size.

## 1 Introduction

As one of the most devastating natural disasters worldwide, drought imposes profound impacts on ecosystems, agricultural systems, and human societies (Choat et al., 2018; Vadez et al., 2024; Ahmadi Lavin et al., 2025). The phenomenon of forest canopy dieback or mortality caused by drought has been widely reported (Greenwood et al., 2017; Preisler et al., 2019; Britton et al., 2022; Helfenstein et al., 2025). Moreover, under climate change, the frequency and intensity of seasonal droughts in humid regions are also expected to increase (Chen et al., 2021; Zhang Y. et al., 2023). For example, in 2022, a severe summer-autumn drought occurred in southern China, leading to widespread plant wilting and death. This has significantly affected the stability and sustainability of forest productivity and carbon sequestration.

Plant water status is a key trait in response to drought and can be characterized using variables such as water potential, hydraulic conductance, and tissue water content. These variables have their advantages and limitations for revealing plant drought response strategies (Sapes et al., 2019). Water potential in xylem and leaves has been extensively employed to quantify water stress in plants. In particular, the water potential at 50% loss of hydraulic conductivity (P_50_) and the hydraulic safety margin (HSM) have emerged as critical metrics for assessing plant vulnerability to drought and the associated risk of mortality (Zhu et al., 2017; Choat et al., 2018; Liang et al., 2021). However, obtaining these hydraulic traits is inherently labor-intensive and time-consuming, and such measurements conducted under laboratory conditions may not capture the actual intensity of drought stress experienced by plants in the field. In response to drought, plants primarily adjust their physiological processes to maintain long-term water balance and to avoid excessive dehydration and desiccation of vital tissues (Bartlett et al., 2016; Gupta et al., 2020). Leaf water content (LWC), therefore, serves as a direct indicator of the degree of tissue dehydration. LWC is widely used to detect plant drought responses and predict drought-induced mortality (Sapes et al., 2019; Konings et al., 2021; Zhou et al., 2021; Trifilò et al., 2023), as LWC is generally easy to measure, and integrates hydraulic failure and carbon depletion mechanisms. For example, Sapes et al. (2019) found that plant water content is a good predictor of drought-induced mortality risk at the population scale, and can differentiate between plants at no risk and those with increasing mortality risk. Similarly, Zhou et al. (2021) demonstrated that LWC exhibits three distinct response phases in LWC dynamics under drought progression, with two critical thresholds marking significant shifts in drought response. Furthermore, previous study has found that the relative water content for tropical species ranged from 16% to 61% at the severely wilted state (Kursar et al., 2009). Given the high variability in LWC under drought environments, greater attention must be paid to leveraging its dynamics to uncover the mechanisms underlying plant responses to drought, because LWC is closely linked to atmospheric drought stress, soil water status, and plant drought resistance (Cao et al., 2015; Yamani et al., 2020; Akter et al., 2023). In fact, plant vulnerability to drought stress is largely determined by the availability of root zone water and how plants utilize the available water. However, there are currently few studies systematically revealing the relationship between leaf water content in plants, soil moisture status, and water-use strategies.

Root water uptake is a key factor influencing plant water status and drought survival, revealing plant adaptation strategies to changes in soil moisture. Numerous studies have shown that plants primarily acquire water from shallow soil layers when soil moisture is sufficient, whereas they progressively shift toward deeper water sources as surface soil desiccates (Eggemeyer et al., 2008; Dai et al., 2023; Yin et al., 2024). This hydraulic plasticity is inherently linked to root architecture depth, with deep-rooted species demonstrating enhanced capacity for water source switching. Moreover, Bello et al. (2019) have revealed that during summer drought, two species (*Quercus petraea* and *Pinus sylvestris*) in pure stands primarily relied on shallow soil water, whereas oak trees in mixed stands were able to utilize deeper water sources. Another study conducted in a subtropical mixed plantation system demonstrated that although three plant species shifted to deeper water sources during drought periods, they consistently competed for water extracted from similar soil depths during both drought and non-drought conditions (Yang et al., 2015). Thus, increasing the uptake of deeper water sources is an important survival strategy for most plants in response to drought (Wang et al., 2017; Ripullone et al., 2020; Bachofen et al., 2024). However, studies conducted in semi-arid and sub-humid forest ecosystems reveal that increased the proportion of deep soil water uptake does not fully alleviate drought stress, as growth still exhibits significant declines (Yin et al., 2024). Liu et al. (2021) have found that plants relying on shallower water sources exhibit higher drought resistance, while species absorbing water from greater depths show lower drought resistance. These findings suggest that the differences in water uptake depth may explain the vulnerability to drought among species, but they also highlight the need for further investigation into the mechanistic linkages between soil water acquisition strategies and tree drought resistance.

Stable isotopes of hydrogen and oxygen have been widely applied in research to identify and quantify the spatiotemporal sources of plant water (Wu et al., 2018; Goldsmith et al., 2022; Luo et al., 2023a; Wang et al., 2023). Plant xylem water represents a mixture of water absorbed by roots from different soil depths. Therefore, based on the assumption of negligible isotopic fractionation during root water uptake, the proportional utilization of water sources from various depths can be estimated by analyzing the isotopic compositions of both xylem water and its potential sources. Thus, various approaches have been developed to estimate the vertical distribution of root water uptake, including the empirical models [e.g., IsoSource (Phillips and Gregg, 2003), MixSIR (Moore and Semmens, 2008), SIAR (Parnell et al., 2010), MixSIAR (Stock et al., 2018)] and process-based models [isoRHEA (Yamanaka et al., 2017), EcH_2_O-iso (Kuppel et al., 2018)]. Among these models, the MixSIAR model is the most widely used due to its simplicity and relatively reliable outcomes. In addition to determining the vertical distribution of root water uptake, isotope-based methods can also track the temporal origin (residence time or water age) of water taken up by plants (Brinkmann et al., 2018; Sprenger et al., 2019; Luo et al., 2023a), shedding light on moisture recharge processes in the root zone and how they respond to precipitation changes. As such, stable isotope analysis of plants and their potential water sources provides strong insights into how plants adapt to environmental changes. Meanwhile, numerous studies have traced shifts in water uptake sources for herbs, shrubs, and trees during drought progression (Prechsl et al., 2015; Ripullone et al., 2020; Liu et al., 2021). However, most research has focused on arid and semi-arid regions, while investigations into the water uptake strategies of shrubs in subtropical humid regions during drought remain limited.

In this study, the soil water dynamics, stable isotope compositions of soil water and xylem water, and plant traits (e.g., size, leaf water content) were observed for two shrubs to investigate their adaptation strategies to drought in the southern China. The main objectives were to (1) compare the differences of leaf water content and root water uptake depth between the two shrubs, and (2) explore the drought vulnerability and its key drivers in two shrub species.

## 2 Materials and methods

### 2.1 Study site and plant materials

This study was conducted in a hillslope located in Dongkou county, Hunan Province (26°51’38”-27°22’23”N, 110°8’40”-110°57’10”E, **Supplementary Figure S1**). This area has a subtropical monsoon climate, with four distinct seasons and abundant heat resources. The average annual air temperature is about 16.6 °C, with an average annual precipitation of 1491 mm. Precipitation is abundant from late spring to early summer, while drought periods frequently occur during mid-summer to early autumn. The predominant soil types in this region are yellow soil, with limestone distributed beneath or on parts of the ground. Vegetation covers almost 80% of the study area, and the dominant plant species comprise trees (*Cryptocarya chinensis*, *Cladrastis platycarpa*) and shrubs (*Vitex negundo*, *Lithocarpus polystachyus*).

Experiments were performed in June, August and October 2022 on two mature shrub species (*V*. *negundo*, *L*. *polystachyus*) under field conditions. The selected shrubs are two native and universal species in this area, characterized by distinct root distribution patterns: *V*. *negundo* is shallow-rooted, while *L*. *polystachyus* is deep-rooted. The observed maximum rooting depth was less than 50 cm for *V*. *negundo* and 60–80 cm for *L*. *polystachyus*. To compare their drought response patterns, three experimental plots (20 m × 20 m) were established on hillslopes with similar slope positions (mid-slope), approximately 50 m apart from each other. Vegetation surveys, including basic measurements of stem basal diameter, plant height, and crown width, were also conducted within these plots.

### 2.2 Measurements of leaf water content

Within each plot, six healthy individuals per shrub species were selected as the target sample plants. 5–10 intact leaves from multiple branches in the upper canopy of each plant were collected. The leaves were sealed in zip-lock bags (to prevent water loss), labeled, placed in a cooler box, and quickly transported to the laboratory (about 2 h) for subsequent measurements. To assess drought impact on plants, 27 individuals per shrub species were selected for leaf sampling in October 2022. In addition, we quantified drought-induced canopy damage degree based on the proportion of canopy leaves exhibiting withering or yellowing.

Fresh weight (W_f_) measurements of leaves were immediately conducted upon their return to the laboratory. Then, dry weight (W_d_) was determined by over-drying samples at 105 °C for 30 min to kill biological activity and then at 80 °C for 48 h to constant weight. Thus, leaf water content (LWC, %) can be calculated as: LWC=(W_f_ -W_d_)/W_f_.

### 2.3 Water samples collection and isotopic analyses

Plant twigs and soil samples were collected on June, August and October in 2022. For each target plant, three parallel samples of twigs were collected from sun-exposed crown using branch shears. The bark and phloem tissue for each twig were immediately removed and cut into 2–3 cm pieces, and put into glass vials (15 ml), sealed with parafilm. Then, these samples were placed in a portable incubator with ice cubes, and brought back to the laboratory for further measurements.

Soil samples were collected near the target plants on the same day. A soil auger was used to obtain the soil cores. Soil samples were taken at 10 cm intervals from 0 to 50 cm depth. For depths greater than 50 cm, only one soil sample mixed from 60 to 80 cm depths was collected, depending on the maximum drilling depth (which varied between 60 and 80 cm). Similarly, part of soil sample from each depth was placed into the glass vials, sealed with parafilm, and then these vials were placed in a cooler box, and brought back to the laboratory for water extraction to measure the hydrogen and oxygen stable isotopic compositions. The remaining soil samples were collected into zip-lock bags for soil water content measurements in the lab.

The twig and soil samples were used to extract water using a cryogenic vacuum distillation system (LI-2100, LICA, Beijing, China). The isotopic compositions (δ^2^H and δ^18^O) of xylem water and soil water were measured by a liquid water isotope analyzer (DLT-100, LGR Inc.). The accuracy of the instrument was ±0.6 ‰ for δ^2^H and ±0.2 ‰ for δ^18^O. Moreover, organic materials in the extracted water may cause errors in isotopic values, and thus the correction was carried out according to the method used in the literature (Luo et al., 2019).

### 2.4 Data collection for precipitation and soil water content

To reveal the degree of the drought stress at the study site, monthly precipitation data were downloaded from the data set of 1-km monthly precipitation dataset for China (1901-2023), which is provided by National Tibetan Plateau Data Centre (https://doi.org/10.5281/zenodo.3114194) (Peng et al., 2019; Peng, 2020). Then, monthly precipitation between 2020 and 2023 corresponding to the study site were retrieved from the dataset.

For soil water content (SWC, cm^3^/cm^3^), the collected soil samples from the plots at varying depths were measured for soil bulk density and SWC using the oven-drying method (24 h at 105°C). Additionally, to quantify soil moisture stress during drought periods, we obtained daily 0–100 cm volumetric soil water content data of 2022 for the study site from the dataset of a 1 km daily soil moisture dataset over China based on *in-situ* measurement (2000-2020). This dataset is provided by National Tibetan Plateau Data Centre (https://doi.org/10.11888/Terre.tpdc.272415) (Li et al., 2022; Shangguan et al., 2022). Based on the daily SWC, soil relative extractable water (REW) was further calculated as: REW=(SWC-SWC_min_)/(SWC_max_-SWC_min_). SWC_max_ and SWC_min_ is the maximum and minimum soil water content observed at the site in 2022, respectively. When the value of REW is less than 0.4, it usually indicates the presence of significant soil water stress (Breda et al., 1995; Luo et al., 2023b).

### 2.5 Data analysis

The source contribution of the different soil depths to root water uptake was estimated using the MixSIAR Bayesian mixing model (Stock and Semmens, 2016). This model incorporates uncertainties related to multiple sources and discrimination factors. The isotopic compositions (δ^2^H and δ^18^O) of the two shrubs were used as mixture data inputs into MixSIAR. The averages and standard errors of isotopic values of plant xylem water and soil water at various depths (0-10, 10-20, 20-30, 30-40, 40-50, and 50–80 cm) were input into the model as “mixture” and “source”, respectively. Here, soil water was the sole input to the mixing model, given that groundwater was inaccessible to plants within the study plots (groundwater table depth >10 m). The running length of the Markov Chain Monte Carlo was set to “long” (Chain Length=300000, Burn-in=200000, Thin=100, Chains=3). The Gelman-Rubin diagnostics (< 1.05) and Geweke diagnostics (|Z| < 1.96) were employed to evaluate model convergence. This was a prerequisite for accepting the results.

Statistical analyses were conducted using Origin2024 software (OriginLab, USA) to perform the basic statistical data of leaf water content and its influencing factors. One-way ANOVA and independent sample *t*-tests were employed to detect significant differences of leaf water content between the two shrubs. Linear regression analysis was performed to quantified the relationships between leaf water content, soil water content and basal dimeter. In addition, the nonlinear association between leaf water content and drought-induced canopy damage degree was modeled using a Logistic Function. Goodness-of-fit for all these analyses was evaluated using the coefficient of determination (R^2^) and root mean squared error. The significance threshold was uniformly set at *P* < 0.05. All the above statistical analyses were used to explore the links between different water acquisition strategies revealed by the MixSIAR model and plant drought responses (characterized by canopy damage and changes in LWC).

## 3 Results

### 3.1 Variations in precipitation, soil water content, and drought stress

**Figure 1:**
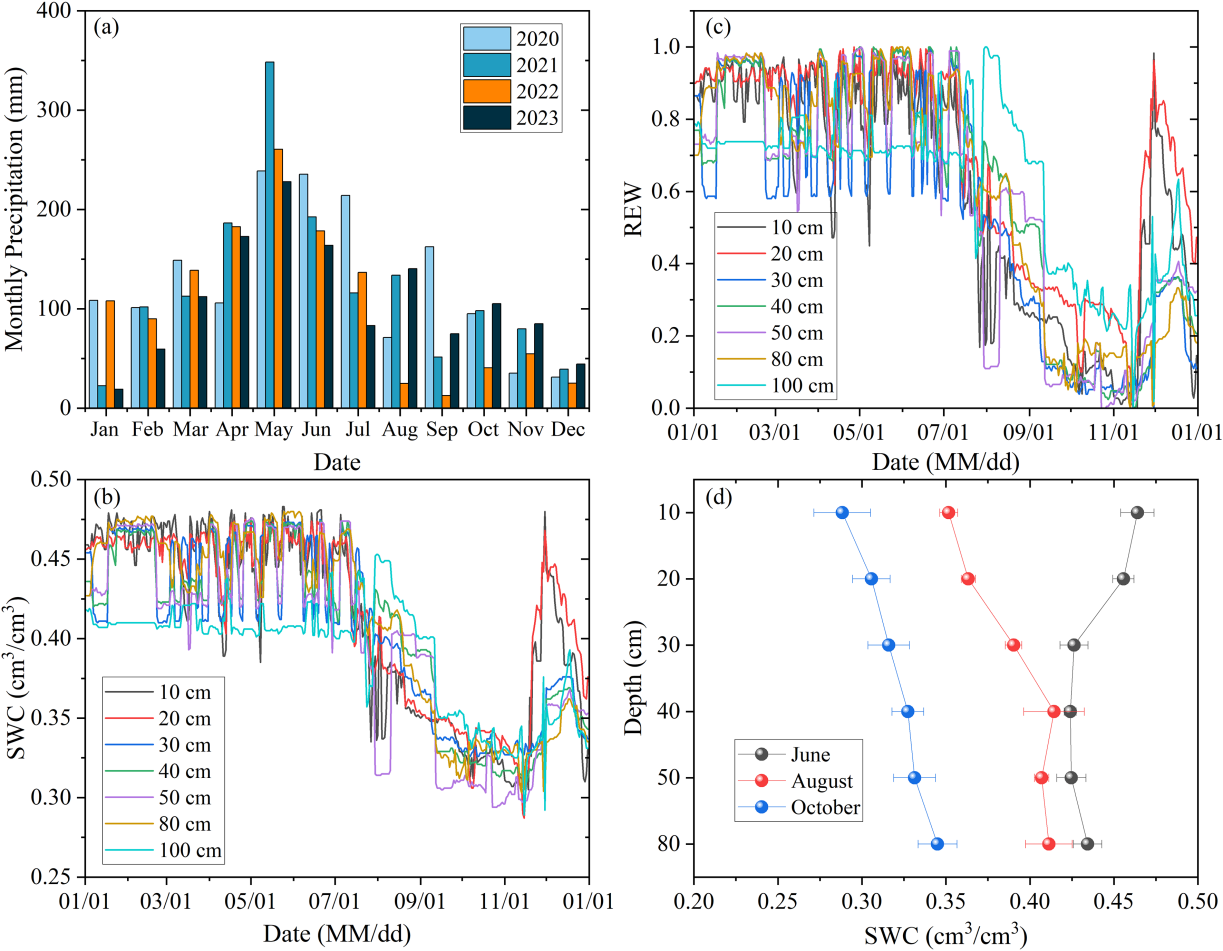
Variations in monthly precipitation between 2020 and 2023 **(a)**, daily soil water content [SWC, **(b)**] and daily relative extractable water [REW, **(c)**] at different depths in 2022. The comparison of SWC observed at various depths at the study site is shown in **(d)**.

For the study area, the average annual precipitation from 2020 to 2023 was about 1393 mm (**Figure 1a**). Although the annual precipitation in 2022 was approximately 1253 mm, monthly precipitation exhibited pronounced seasonal variability. Specifically, precipitation between August and October was significantly lower than that in the same period of other years, with the total rainfall during these months declining by 72% to 76% compared to that in other years. This seasonal variability in precipitation also drove dis-tinct seasonal fluctuations in soil moisture at different depths, with the surface layer (0–30 cm) exhibiting a stronger response to rainfall than deeper soil layers (**Figure 1b**). During the study period in 2022, soil moisture ranged from 0.29 to 0.48 cm³/cm³, with slight variations across depth intervals.

A severe drought event spanning summer and autumn occurred in 2022. Exceptionally low precipitation from August to October caused soil moisture to decline rapidly (**Figures 1b, d**), triggering acute soil water stress (REW < 0.4, **Figure 1c**). The entire 0–100 cm soil profile experienced varying degrees of water stress, particularly severe in surface layers. This extreme drought persisted for over three months, with maximum intensity occurring between late October and early November. Although rainfall in November temporarily alleviated water stress in the top 20 cm of soil, moisture deficits below 30 cm depth continued. This indicates that recovery from drought may require additional substantial precipitation.

### 3.2 Changes in leaf water content and drought response

**Figure 2:**
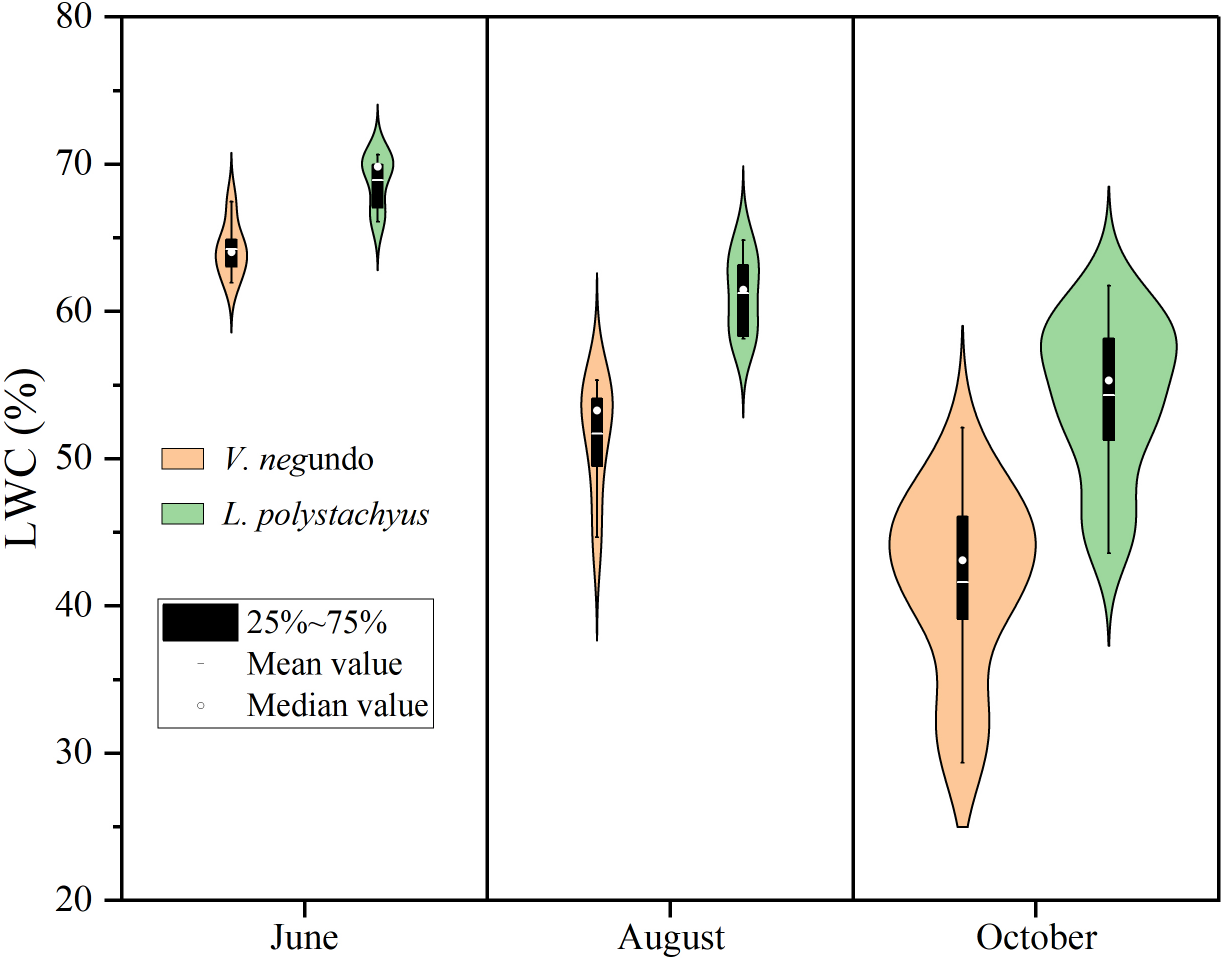
Violin plots showing the distribution of leaf water content (LWC) and their variations with time. The mean values show significant differences (P< 0.05) both at different times and between the two species.

**Figure 2** illustrates different responses of LWC of the two species during the severe drought. The LWC of the two shrubs decreased significantly (*P* < 0.05) during the drought progression. Specifically, *V. negundo* exhibited a reduction from 64.2% on June to 41.6% on October, while *L. polystachyus* declined from 68.9% to 53.7%. Notably, the mean LWC of *L. polystachyus* was significantly (*P* < 0.05) higher than that of *V. negundo.*

**Figure 3:**
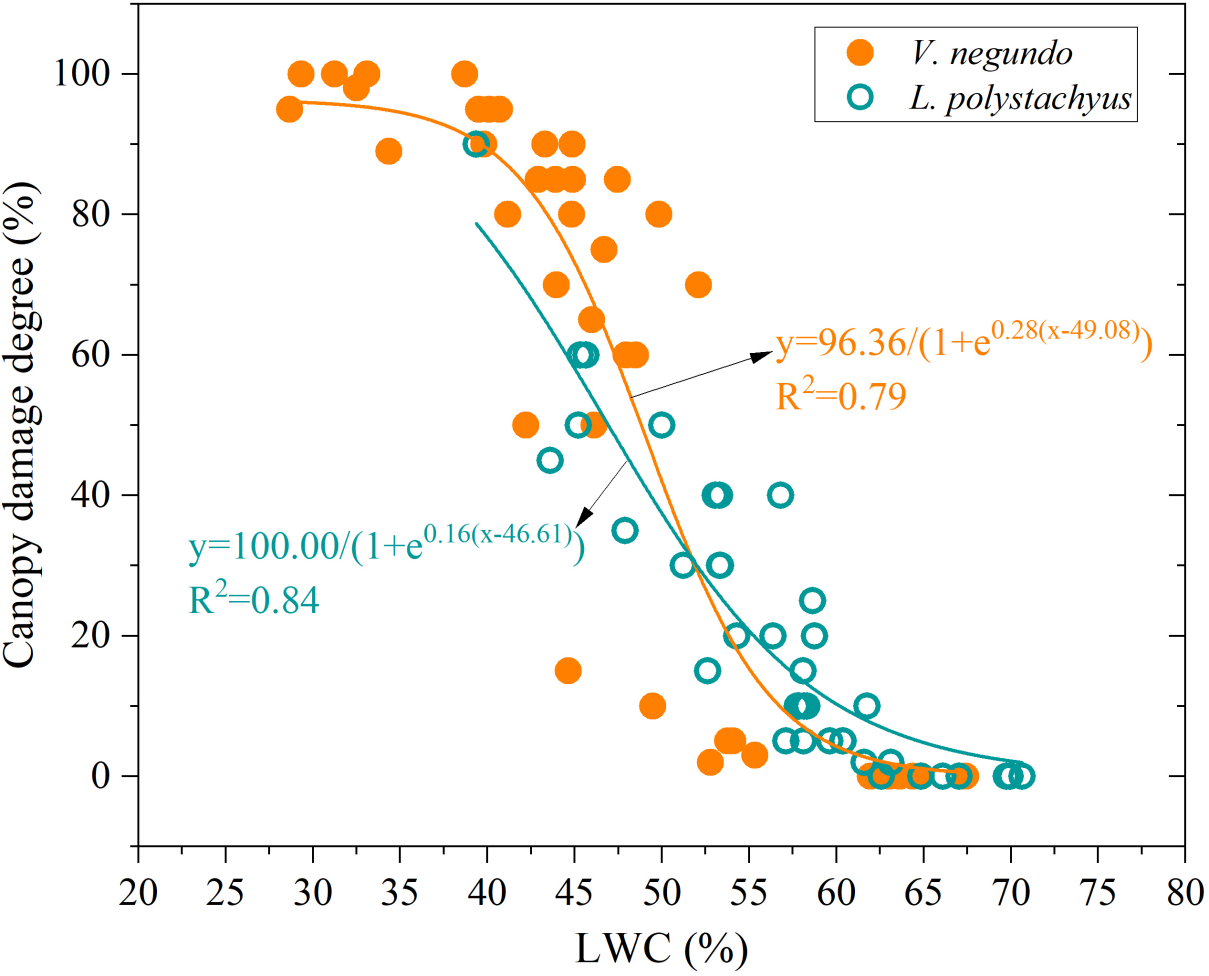
The relationship between leaf water content (LWC) and drought-induced canopy damage degree.

Changes in LWC effectively reveal different drought responses and damage characteristics (**Figure 3**). A significant nonlinear relationship (sigmoidal curve) emerged between LWC and canopy damage severity. When LWC exceeded 60%, minimal canopy damage (<10%) occurred. Conversely, when LWC fell below 45%, canopy damage exceeded 70%, with extensive leaf chlorosis and dieback observed. Moreover, *V. negundo* showed significantly elevated drought-induced canopy damage (*P* < 0.05) compared to *L. polystachyus*, with different drought sensitivity between the two shrubs (reflected by the parameters of the fitting equation, **Figure 3**). This indicates that *V. negundo* has a higher vulnerability to drought.

### 3.3 Variations in root water uptakes

**Figure 4:**
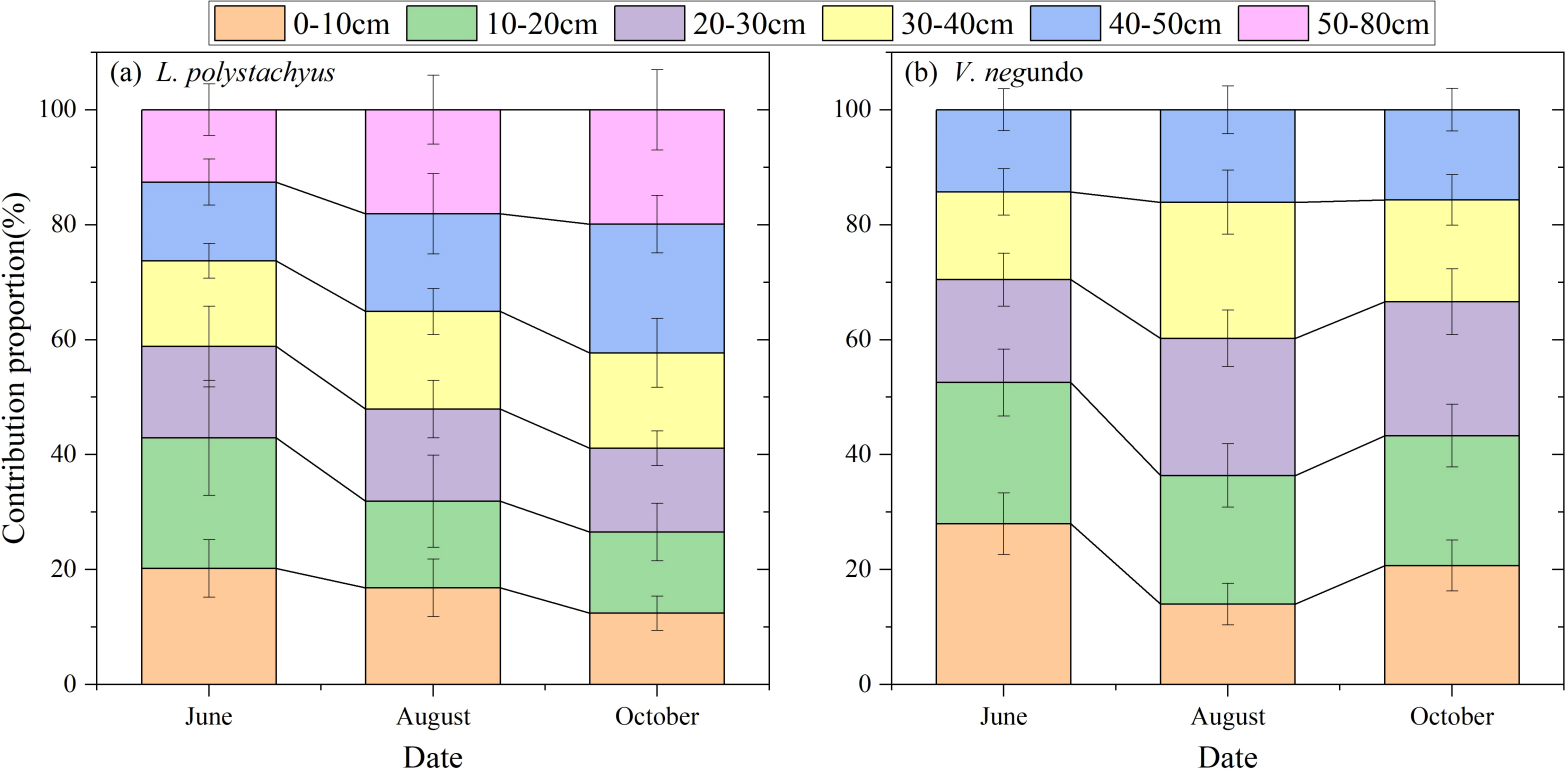
Variations in the relative contribution proportions of potential water sources from different soil depths for *L. polystachyus* **(a)** and *V. negundo* **(b)**.

The two shrubs exhibited distinct water utilization strategies. During the relatively wet period in June, *L. polystachyus* derived 58.8%, 28.6%, and 12.6% of its water sources from shallow (0–30 cm), intermediate (30–50 cm), and deep (>50 cm) soil layers, respectively (**Figure 4a**). In contrast, *V. negundo* primarily relied on shallow water sources (70.5%), while intermediate layers contributed only 29.6%, and deep-layer water uptake was absent as their maximum rooting depth < 50cm. (**Figure 4b**).

During the early drought period (August), as shallow soil moisture decreased significantly (**Figure 1**), both species substantially reduced shallow water exploitation while markedly increasing water uptake from intermediate soil layers (34% and 39.8% respectively). Meanwhile, *L. polystachyus* elevated deep-layer water utilization to 18.1%. During the severe drought in October, *L. polystachyus* further increased intermediate (39%) and deep-layer (19.9%) water exploitation (**Figure 4**). However, *V. negundo* relied predominantly on shallow soil water (66.6%). The above results indicate that (soil) drought obviously influences the proportion of water uptake by roots, and such different water use strategies of the two shrubs were closely related to their drought vulnerability, where increasing the proportion of deep water use contributes to their drought tolerance.

### 3.4 The influencing factors on drought vulnerability

**Figure 5:**
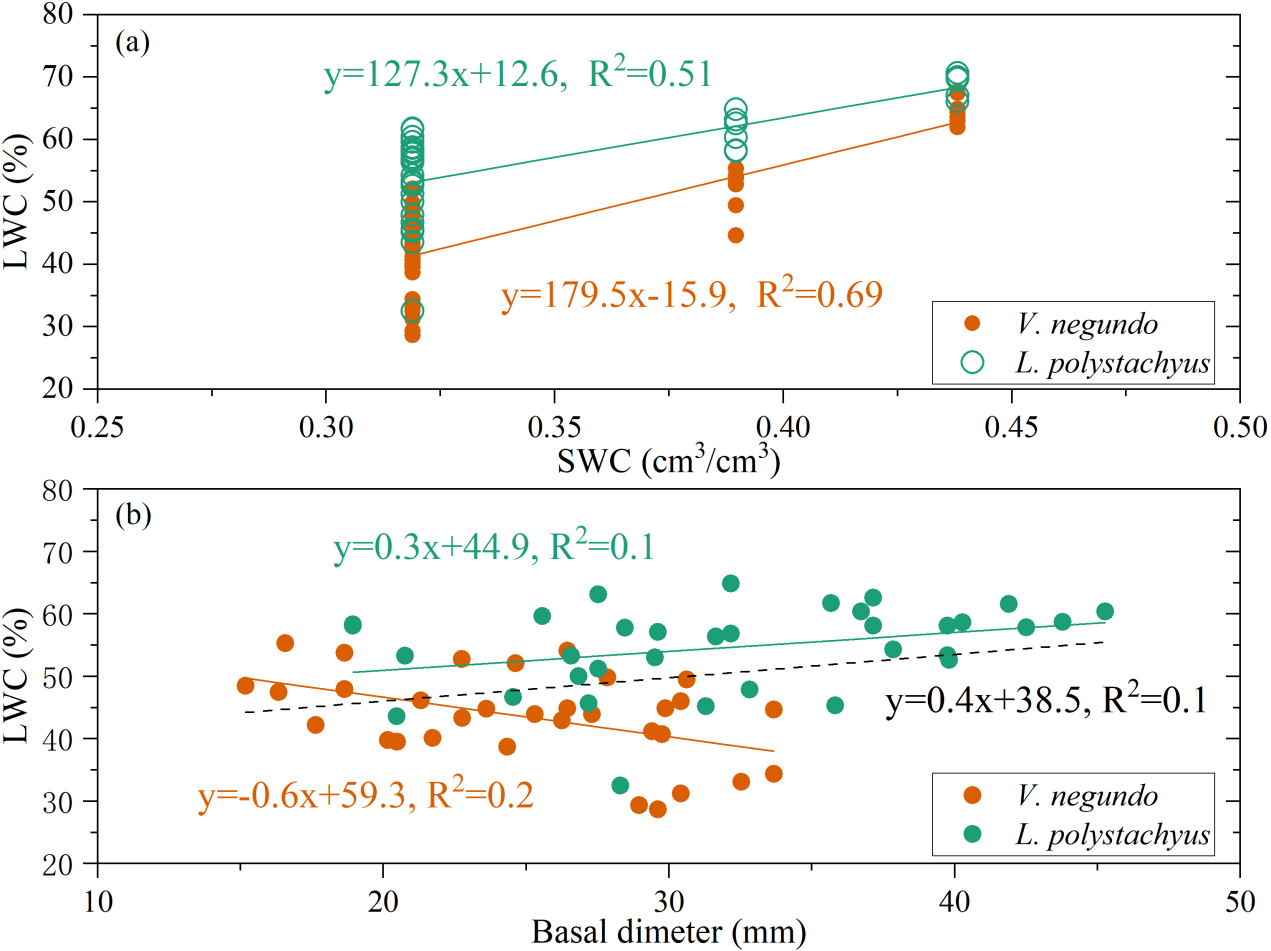
The relationship of leaf water content (LWC) and soil water content [SWC, **(a)**], LWC and basal dimeter **(b)** for the two species during drought period.

Variations in LWC were primarily mediated through modification of plant-available moisture conditions. **Figure 5a** demonstrates a statistically significant positive correlation between LWC and soil water content (SWC) (*P* < 0.05), accounting for 51% and 69% of LWC variability for *L. polystachyus* and *V. negundo*, respectively. Overall, LWC exhibited a significant positive relationship with stem basal diameter (*P* < 0.05). However, the responses of LWC to basal diameter showed significant different sensitivity between the two species. Such difference is likely related to their water use strategies. Generally, for a given species, larger individuals tend to access water from deeper and more extensive soil layers. However, for *V. negundo*, the majority of its fine roots are concentrated in shallow soil layer. As a result, *V. negundo*, which relies more on shallow water sources (**Figure 4**), inevitably experienced greater water stress during drought periods. A significant negative correlation was observed in *V. negundo* (**Figure 5b**). This indicates that larger individuals of *V. negundo* may exhibit lower LWC under drought stress, thereby exhibiting greater drought vulnerability.

## 4 Discussion

### 4.1 Responses of LWC to soil water availability

Our findings confirm that LWC serves as a sensitive integrator of plant water status responding to drought stress. In this study, LWC showed a significant linear relationship with SWC (**Figure 5**). Such results are consistent with previous studies (Zhou et al., 2021). However, other studies have found that there is a significant nonlinear relationship between the LWC and SWC (Yan et al., 2017; Sun et al., 2024). This is consistent with the theory of nonequivalence, which states that there are significant differences in the effects of soil water on plant growth during drought period (Kramer, 1994). In this study, the relationship between LWC and SWC was linear rather than showing a threshold-type relationship. The observed linear relationship likely reflects the specific soil moisture gradient captured during our monitoring period, and extrapolation beyond this range should be made with caution. Anyway, these studies demonstrate that soil water conditions exert a significant controlling influence on plant water status.

Soil water availability often exhibits significant temporal and spatial variations. Interspecific variations in leaf water content are largely governed by vertical heterogeneity in root-zone soil moisture and species-specific root water uptake capacities (Zhou et al., 2021; Trifilò et al., 2023; Li et al., 2025). In this study, when soil moisture at various depths was generally sufficient, the difference in leaf water content between the two shrubs remained minimal due to adequate water accessibility. However, during drought periods, as topsoil moisture decreased drastically, the LWC of *L. polystachyus* (reliant on deep-layer water sources) was significantly higher than that of *V. negundo* (depended on shallow soil water). Recent study on *Pinus massoniana* in subtropical humid regions had found that the reliance on shallow water positively correlated with water-use efficiency via negatively influencing LWC and stomatal conductance (Li et al., 2025). Furthermore, on a larger scale, Wang R. et al. (2021) have examined the LWC variations of 5641 plant species across diverse climatic zones in China, revealed considerable variability in LWC among plants from different soil conditions. Notably, they observed a decrease in LWC in humid regions and an increase in dry environments. This variation in LWC across species and climatic zones reflects long-term evolutionary adaptations of plants to their specific soil water conditions.

### 4.2 Influence of plant size on its responses to drought stress

For decades, plant size has been linked to differential drought tolerance (Casper, 1996; Blum and Sullivan, 1997; Wang N. et al., 2021; Deng et al., 2025). In this study, the relationship between plant LWC and drought for the two species was regulated by plant size (basal dimeter in this study), with smaller plants for *L. polystachyus* exhibiting greater vulnerability to drought stress than larger plants. This result aligns with the findings of Wang et al. (Wang N. et al., 2021), suggesting that larger plants possess superior drought resistance capabilities. This advantage may be attributed to their deeper root systems and higher water use efficiency (de Ollas et al., 2023). On the contrary, some studies indicate that larger plants may be more vulnerable to drought stress than smaller ones during drought events (Blum and Sullivan, 1997; Carrer and Urbinati, 2004; Deng et al., 2025). This is in line with the results observed on *V. negundo* in this study. Generally, larger plants possess greater demand for root-zone soil moisture, which may intensify water competition under drought conditions. In addition, larger plants may face greater hydraulic challenges. They possess longer hydraulic pathways from roots to leaves, increasing resistance and the risk of xylem cavitation under water stress (Midgley, 2003; Salguero-Gomez and Casper, 2011). This can contribute to the earlier onset of LWC decline. Therefore, the capacity of larger plants to cope with drought is context-dependent. They may be more resistant to mild or moderate stress due to their adaptations, but can become more vulnerable under extreme stress due to their high resource demands. Over all, the relationship between plant size and drought tolerance is not monolithic but varies with drought intensity and duration.

Furthermore, our results revealed that although the base diameter was similar, *V. negundo* displayed higher drought sensitivity than *L. polystachyus*. This finding is not surprising, as the root water uptake strategies between the two species were significantly different. Thus, this further leads to the fact that they actually experienced different levels of drought stress. Of course, this study focused exclusively on two shrubs. It is evident that divergent physiological adaptations-such as variations in root distribution depth and stomatal regulation capacity-likely lead to species-specific responses to drought stress (Kühnhammer et al., 2023; Luo et al., 2023a). Further investigation is required to determine whether analogous patterns of drought response are observed in other plant species.

### 4.3 Implications and limitations

Our results emphasize that variations in root water uptake depth and leaf water content can well explain the differences in drought vulnerability of the two shrubs, which provides a useful framework to evaluate belowground constraints on water resource acquisition and plant water status for drought responses. In the context of climate change-induced increases in the frequency and intensity of droughts, the drought response of trees in forest ecosystems and their mortality patterns are of wide interest (Allen et al., 2010; Luo et al., 2016; Greenwood et al., 2017; Cheng et al., 2024), probably due to the importance of tall trees in forest systems and the ease with which tree mortality over larger areas can be captured by remote sensing-based methods (Liu et al., 2022). Shrubs, on the other hand, are often scattered between the tree layers or under the canopy, and it is difficult to investigate responses of shrub to drought based on remote sensing methods. However, shrubs play an important role in forest diversity, as species diversity has been found to significantly increase the drought tolerance of forests (Fichtner et al., 2020; Zhang, T. et al., 2023; Bazzichetto et al., 2024). Therefore, our study calls for the need to pay more attention to the drought vulnerability of shrubs in the future to lay the foundation for a complete understanding of drought response mechanisms in forest ecosystems.

However, there are some limitations in our study. Firstly, this study only investigated two typical shrubs at the study site, and thus the number of species studied was limited. Although the results of this study help us to understand the response mechanism of plants to drought, future studies should focus on a wider range of plant species to reveal community-level drought response characteristics. Secondly, soil texture was not explicitly measured across plots, and its specific effect remains uncertain. However, the core effects of soil texture on plant available water are mediated through soil moisture. By directly monitoring soil water content, our study design captured this critical variable, meaning the functional implications of soil texture were already accounted for in our analysis of drought response. Thirdly, we found that both species increased the proportion of water utilization in the middle soil layer at the beginning of the drought, which may increase the competition for water in this layer. Although we estimated the proportion of water uptake at different depths, we have not yet quantified the amount of water uptake at different depths in this study, which constrained our quantitative characterization of interspecies water competition relationships. This is a common challenge associated with the widely used stable isotope approach. Water competition among species may directly affect their drought vulnerability (Pérez-Anta et al., 2024; Jiang et al., 2025), especially in forest ecosystems with high species diversity. Investigating of water competition or sharing relationships among species is an important and urgent research topic in this field. However, since quantitatively analyzing interspecific water competition was not a primary objective of this study, this limitation does not undermine the credibility of our core findings regarding the divergent water use strategies and drought vulnerability of the two species.

## 5 Conclusions

This study examined the interspecific differences in the leaf water content and root water uptake patterns during the extreme drought in 2022. LWC serves as a robust drought indicator, capturing the integrated effects of meteorological drought, soil water supply capacity, and plant water uptake strategy. Our findings demonstrate that root water uptake depth governs the LWC variability and drought vulnerability between the two shrubs. During the wet period (June), both shrubs utilized water primarily from shallow soil layers (0–30 cm), with contributions of 58.8% for *L. polystachyus* and 70.5% for *V. negundo*. The belowground niche partitioning of the two shrubs in water uptake existed during the drought period. *L. polystachyus* was able to shift water uptake toward intermediate (39%) and deep soil layers (19.9%), while *V. negundo* relied more on water sources in shallow soil layers (66.6%).

Meanwhile, the relationship between canopy damage severity and LWC for the two species exhibited a significantly fitted sigmoidal curve (R^2^ > 0.8, *P* < 0.05). When LWC fell below 45%, canopy damage exceeded 70%, with extensive leaf chlorosis and dieback observed for *V. negundo*. In addition, larger individuals of *V. negundo* showed greater drought vulnerability (linked to reliance on shallow soil water), their higher soil moisture demand and hydraulic limitations may increase vulnerability during prolonged drought. In the context of climate change, plants in subtropical humid forests are increasingly compromised by extreme drought. The findings in this study may provide mechanistic basis for monitoring and prediction of plant water status and drought vulnerability under water stress conditions.

## Data Availability

The original contributions presented in the study are included in the article/Supplementary Material. Further inquiries can be directed to the corresponding author.
