# Supplementary material for: Leaf water content and water source partitioning reveal species-specific drought vulnerabilities in subtropical shrubs
Source: Front Plant Sci. 2025 Oct 28;16:1684521. doi: 10.3389/fpls.2025.1684521 (PMC12602465; doi:10.3389/fpls.2025.1684521)
Supplement: Supplementary file 1 [file DataSheet1.pdf]

Leaf water content and water source partitioning reveal species-specific drought vulnerabilities in subtropical shrubs

Wenxi Peng[1], Bo Jiang[1], Leru Chang[2], Zidong Luo[3,4*]

[1]School of Economics and Management, Hunan Open University, Changsha 410004, China

[2]Department of Eco-culture and Eco-Tourism, Hunan Vocational College of Engineering, Changsha 410151, China

[3]Institute of Subtropical Agriculture, Chinese Academy of Sciences, Changsha 410125, China

[4]Guangxi Key Laboratory of Karst Ecological Processes and Services, Huanjiang Observation and Research Station of Karst Ecosystems, Chinese Academy of Sciences, Huanjiang 547000, Guangxi, China

*** Correspondence:**

Zidong Luo

zidong.luo@isa.ac.cn

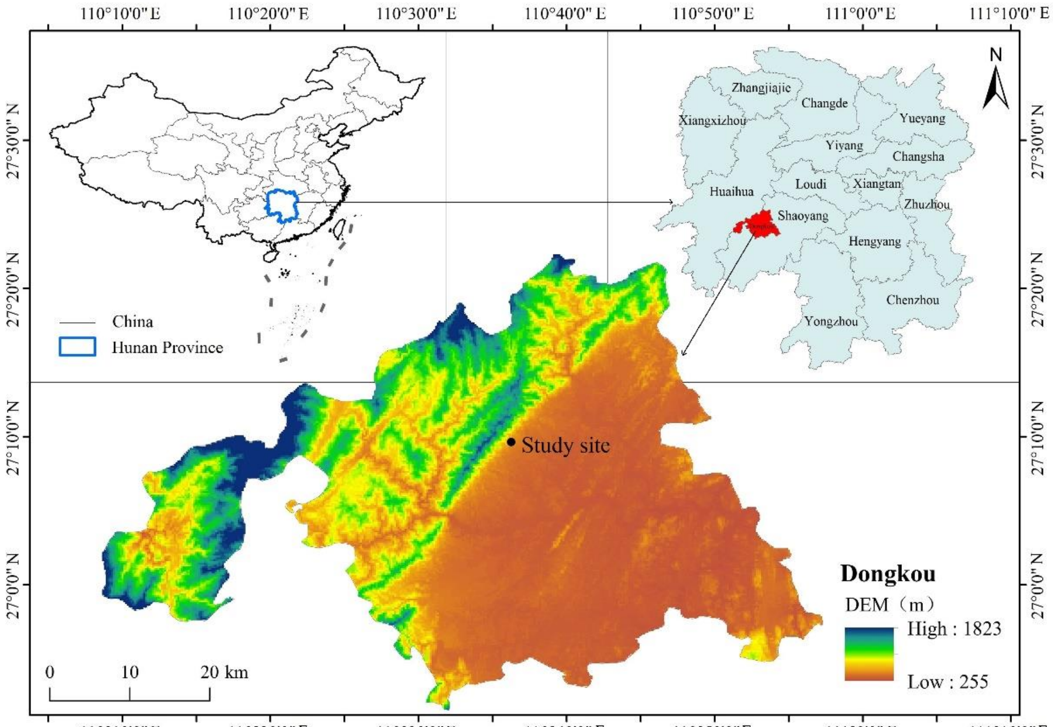

Figure S1 Location of study site.
